# Acoustic resolution photoacoustic Doppler flowmetry for assessment of patient rectal cancer blood perfusion

**DOI:** 10.1117/1.JBO.29.S1.S11517

**Published:** 2024-01-13

**Authors:** Sitai Kou, Xiandong Leng, Hongbo Luo, Haolin Nie, Quing Zhu

**Affiliations:** a Washington University in St. Louis, Department of Biomedical Engineering, St. Louis, Missouri, United States; b Washington University in St. Louis, Department of Electrical and System Engineering, St. Louis, Missouri, United States; c Washington University School of Medicine, Department of Radiology, St. Louis, Missouri, United States

**Keywords:** acoustic resolution photoacoustic microscopy, photoacoustic Doppler flowmetry, ultrasound, rectal cancer, endoscopy

## Abstract

**Significance:**

Photoacoustic Doppler flowmetry offers quantitative blood perfusion information in addition to photoacoustic vascular contrast for rectal cancer assessment.

**Aim:**

We aim to develop and validate a correlational Doppler flowmetry utilizing an acoustic resolution photoacoustic microscopy (AR-PAM) system for blood perfusion analysis.

**Approach:**

To extract blood perfusion information, we implemented AR-PAM Doppler flowmetry consisting of signal filtering and conditioning, A-line correlation, and angle compensation. We developed flow phantoms and contrast agent to systemically investigate the flowmetry’s efficacy in a series of phantom studies. The developed correlational Doppler flowmetry was applied to images collected during *in vivo* AR-PAM for post-treatment rectal cancer evaluation.

**Results:**

The linearity and accuracy of the Doppler flow measurement system were validated in phantom studies. Imaging rectal cancer patients treated with chemoradiation demonstrated the feasibility of using correlational Doppler flowmetry to assess treatment response and distinguish residual cancer from cancer-free tumor bed tissue and normal rectal tissue.

**Conclusions:**

A new correlational Doppler flowmetry was developed and validated through systematic phantom evaluations. The results of its application to *in vivo* patients suggest it could be a useful addition to photoacoustic endoscopy for post-treatment rectal cancer assessment.

## 1 Introduction

Rectal cancer is prevalent in the United States, with an estimated 43,340 new cases in 2020, and the incidence rate among people 50 and younger has been increasing for the past decade.^1^ Rectal cancer requires complex care coordinated among surgeons, oncologists, and radiation specialists to achieve the highest survival rate. In recent years, clinical trials have investigated a non-operative strategy called “watch and wait,” which allows patients with complete clinical responses to treatment to avoid surgical rectal resection and still achieve good long-term oncologic and functional outcomes.^2^^,^^3^ “Watch and wait” has gained tremendous interest, particularly among patients wishing to avoid often highly morbid rectal surgery. However, the strategy relies on accurate assessment of tumor regression and confident identification of patients with a pathological complete response (pCR), i.e., no residual tumors. Current assessment tools include digital rectal examination, endoscopy with biopsy, and functional MRI (fMRI). Unfortunately, none of these modalities is particularly sensitive to residual tumor in the post-treatment rectum.^4^^–^^6^

Photoacoustic imaging (PAI) is a promising functional imaging technique that offers tumor vasculature and blood perfusion information.^7^ Utilizing the ultrasound (US) waves created by tissue’s optical absorption of short laser pulses, PAI can map tissue microvasculature patterns as well as blood flow profiles. Photoacoustic microscopy (PAM) is based on the PAI principle and classified as either optical-resolution (OR) PAM or acoustic-resolution (AR) PAM; AR-PAM can penetrate deeper than OR-PAM by using acoustic focusing rather than optical focusing. Recently, we have developed a co-registered AR-PAM and US system (AR-PAM/US) to assess post-treatment rectal cancer patients. It has been shown to offer residual tumor vasculature information valuable for post-treatment assessment of rectal cancer.^8^

Although it provides better resolution and vascular contrast than fMRI, AR-PAM has limitations in quantitatively assessing rectal tissue. The PA signal is the product of local optical fluence and tissue absorption, and optical fluence can vary with tissue depth due to inhomogeneous light absorption and scattering that contribute to changes in the PA signal amplitude.

OR-PAM Doppler was introduced by Fang et al. in 2007.^9^^–^^11^ Since then, several experimental studies using AR-PAM have been introduced, implemented, and evaluated.^12^^,^^13^ A particularly interesting application is the time domain correlational shift Doppler proposed by Brunker et al.^14^ In their work, they demonstrated the potential of calculating flow speed via A-line cross-correlation in the time domain and performing fixed angle correction to estimate the actual speed with solid and linear flow phantoms.^12^ Other methods of obtaining photoacoustic flowmetry information, such as the dual-pulse photoacoustic flowmetry, have also been implemented.^15^ However, the short delay required to take advantage of the Grueneisen relaxation effect renders it very challenging to adapt for AR-PAM application.

In this study, our AR-PAM/US system implements a quantitative time domain cross-correlational Doppler algorithm to extract the flow signal independent of the signal amplitude. To extract blood perfusion information, the flowmetry processing consists of signal filtering and conditioning, A-line correlational processing, and angle compensation. We first validated our implementation with linear and spiral flow phantoms and contrast agents that we developed for AR-PAM imaging, obtaining measurements that agreed well with theoretical values. Then, to assess blood perfusion and correlate it with treatment response, we applied the Doppler algorithm to *in vivo* AR-PAM endoscopy data from patients who were treated with chemoradiation. To the best of our knowledge, this is the first application of AR-PAM Doppler for assessing the treatment responses of rectal cancer patients.

## 2 Materials and Methods

### 2.1 ARPAE/US System

The AR-PAM/US system has been described earlier.^8^ Briefly, the system comprises a 1064 nm Nd: YAG laser (DPS-1064-Q,^16^, China) operating at 1 kHz with pulse energy of ∼8  mJ, an US pulser-receiver, a function generator, a DAQ PC, and an imaging probe. The ultrasound transducer (US XDC) was custom made by Capistro Labs (San Clemente, California), using Polyvinylidene Fluorid (PVDF) material. The US transducer central frequency is 15 MHz with a 115% bandwidth. The US pulser receiver was a Panametric (currently Olympus, Tokyo, Japan) 5900PR, the function generator was a Tektronix (Beaverton, Oregon) AFG1022, and the DAQ was an Alazartech (Pointe-Claire, Quebec, Canada) ATS9462. To develop this system, the probe was modified to use a large 5 mm laser line mirror for better optical and mechanical stability. The function generator generates pairs of pulses with a short 16.3  μs delay: the first triggers the laser to generate a PA pulse, and the second triggers the US pulser-receiver to generate a US pulse. Both signals are amplified by the pulser-receiver before being received by the DAQ PC and digitized at 180 MHz.

**Fig. 1:**
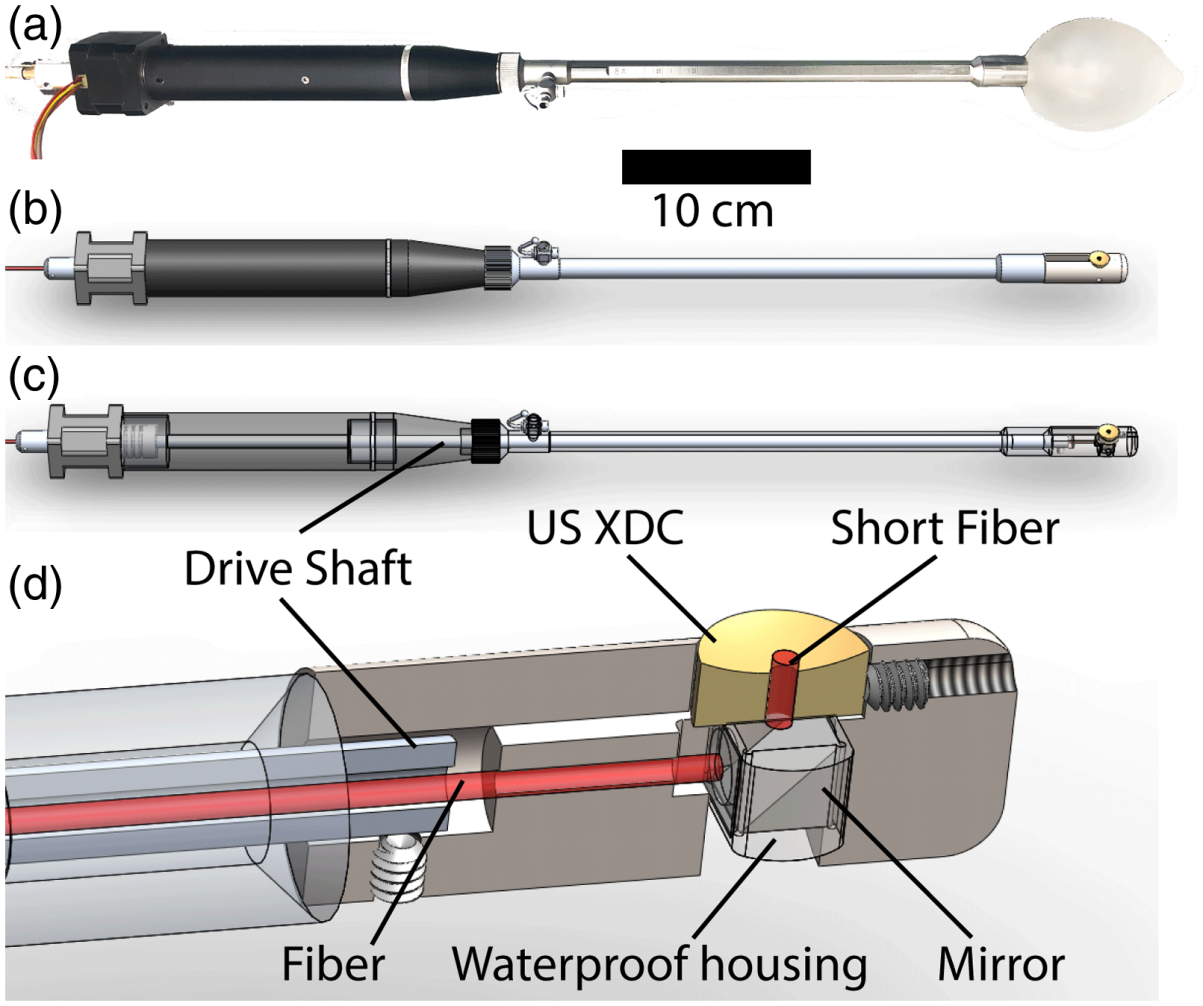
AR-PAM/US system components (a) Probe body picture. (b) Probe body CAD. (c) Probe body with transparent parts. The stepper motor, at the extreme left, turns the drive shaft in small radial increments. The drive shaft and probe body house the US XDC wires (not shown) and the optical fiber, which extends along the center of the probe. (d) Cross-sectional view of the imaging head. The drive shaft is fixed to the imaging head with set screws. The optical fiber passes through a small opening in the waterproof housing holding the laser line mirror that reflects light into the short fiber fixed in the center hole of the US XDC.

Using a LabVIEW (National Instrument, Austin, Texas) code, the DAQ PC also synchronously drives a stepper motor that rotates the imaging head via a drive shaft embedded in the probe, shown in Fig. 1(c). Circular microsteps scan 1000 PA and US A-lines in a full rotation. The system is limited by the laser pulse repetition frequency at 1 kHz, giving a 1 kHz A-line scan rate and an ∼1  Hz B scan rate. The PA and US data obtained from scanning are processed in real time and displayed on the DAQ PC monitor for evaluation. The 6 dB US lateral resolution and axial resolution were characterized by 7  μm diameter carbon fiber targets to be 83 and 30  μm, respectively, at the transducer focus. The PA resolution, characterized with the same target, measured 128  μm laterally and 68  μm axially at the US focal distance.

### 2.2 Spiral Phantom Preparation

**Fig. 2:**
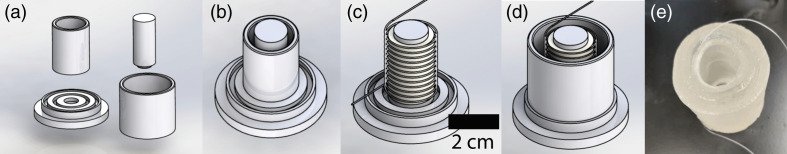
Drawings illustrating the preparation of a spiral flow phantom. (a) Individual molds. (b) Mold assembly for making a small cylindrical phantom. (c) Resulting small cylindrical phantom with spiral tubing on its exterior. (d) Mold with sleeve for encasing the spiral tubing to produce the final phantom. (e) Image of completed spiral flow phantom with embedded tubing.

A tissue-mimicking phantom with embedded spiral tubes was produced to simulate the imaging setting of a colorectal tissue scan with blood flow. Specifically, a two-pour approach and assemblies of three-dimensional (3D) printed phantom molds shown in Fig. 2(a) were used to first produce a tubular gelatin phantom and then to embed a spiral of tubing on the outside of the phantom. The phantom was made of 8%w gelatin solubilized in degassed deionized (DI) water to match the speed of sound in biological tissue’s while limiting optical absorbance. To retard degradation of the phantom, 1%v Germall Plus preservative was added.^17^ This mixture was degassed in a vacuum chamber to remove air bubbles formed during mixing. After the mold was configured as in Fig. 2(b), the gelatin mixture was added and allowed to cool to 10°C, forming a small hollow cylindrical phantom. Then, PTFE tubing with a 580  μm ID was spiraled onto the phantom [Fig. 2(c)]. The outer wall mold is mounted around the phantom, as shown in Fig. 2(d), then the gelatin mixture is poured into the mold and allowed to cool to give the final spiral flow phantom shown in Fig. 2(e).

### 2.3 Doppler Processing

Doppler flowmetry in the phantom and in tissue utilizes a correlation Doppler algorithm with extensive noise rejection features. The PA signal is first passed through an FIR filter with a 3 to 30 MHz passband, then the average intensity of the noisy regions is analyzed and used to apply amplitude-based thresholding to the signal to avoid noise correlation. The signals are interpolated by a factor of 3 to allow finer correlation shift calculation as:

$$V_{\mathrm{res}}=c/2*f_{\mathrm{prf}}/(f_{s}*n_{\mathrm{res}})=1/2*1540\;m/s*1\;\mathrm{kHz}/180\;\mathrm{MHz}/3=1.42\;\mathrm{mm}/s,$$ (1)

where $V_{\mathrm{res}}$ is the velocity resolution, c is the speed of sound in the medium, $f_{\mathrm{prf}}$ is the pulse repetition frequency of the laser, $f_{s}$ is the sampling frequency, $n_{\mathrm{res}}$ is the signal interpolation factor.

For adjacent A-lines, a 25-point sliding window cross-correlation is calculated, and their correlation shift and correlation amplitude are recorded:

$$V=V_{c}/\cos\;\theta =P_{\text{shift}}*V_{\mathrm{res}}/\cos\;\theta ,$$ (2)

where V is the actual velocity, $V_{c}$ is the velocity in A-line direction, θ is the angle between flow direction and A-line direction, $P_{\text{shift}}$ is the shift in pixels calculated by cross-correlation.

The correlation amplitude is used to filter out remaining noisy correlations. After filtering, the correlation shift can be converted to a velocity map based on Eqs. (1) and (2).^18^ Equation (1) calculates the Doppler velocity resolution, $V_{\mathrm{res}}$. Because the direction of the scanning and the direction of the flow are at an angle relative to one another, angle compensation is added to the velocity calculation by using Eq. (2).

**Fig. 3:**
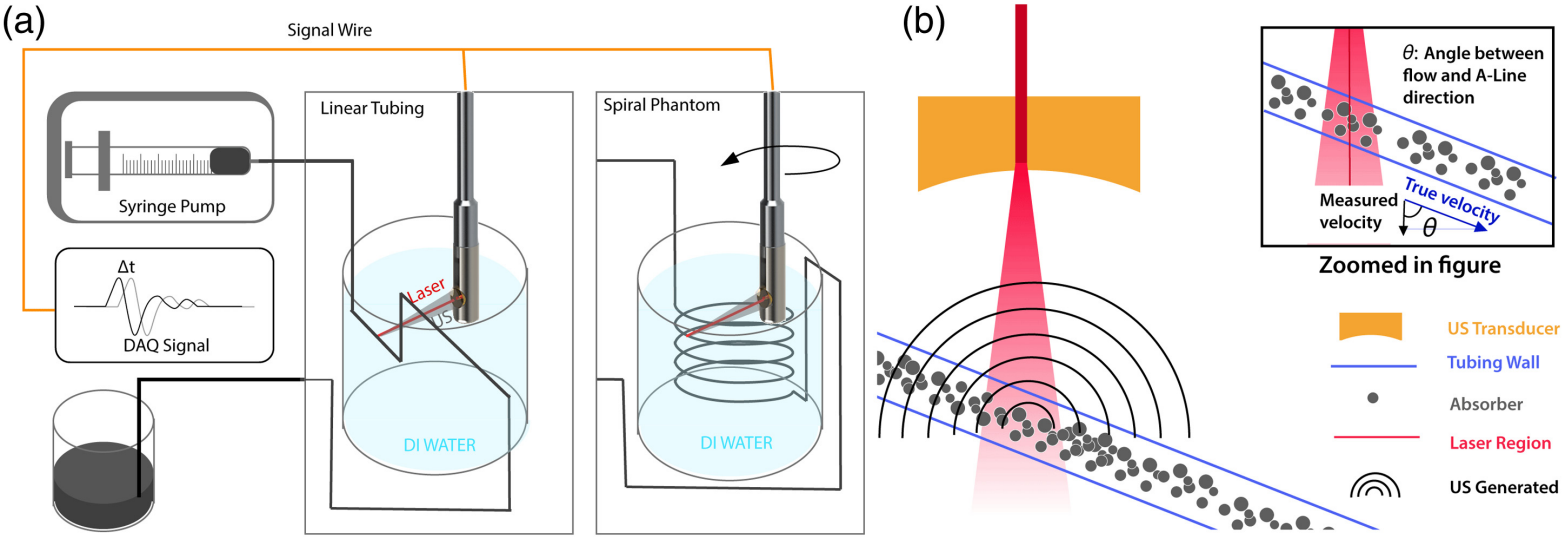
(a) Experimental flow phantom setup with linear tubing suspended in DI water (left) and with spiral tubing gelatin phantom fixed in DI water (right). Contrast agents suspended in DI water flow from the syringe pump through the tubing, which is imaged by the endoscopic imaging probe. (b) Illustration of the linear tubing being scanned. Upon laser exposure, optical contrast agents traveling in the tubing generate PA signals that are picked up by the US XDC. Angle θ for angle correction between the true velocity direction and measured velocity direction is illustrated in the zoomed-in figure.

**Fig. 4:**
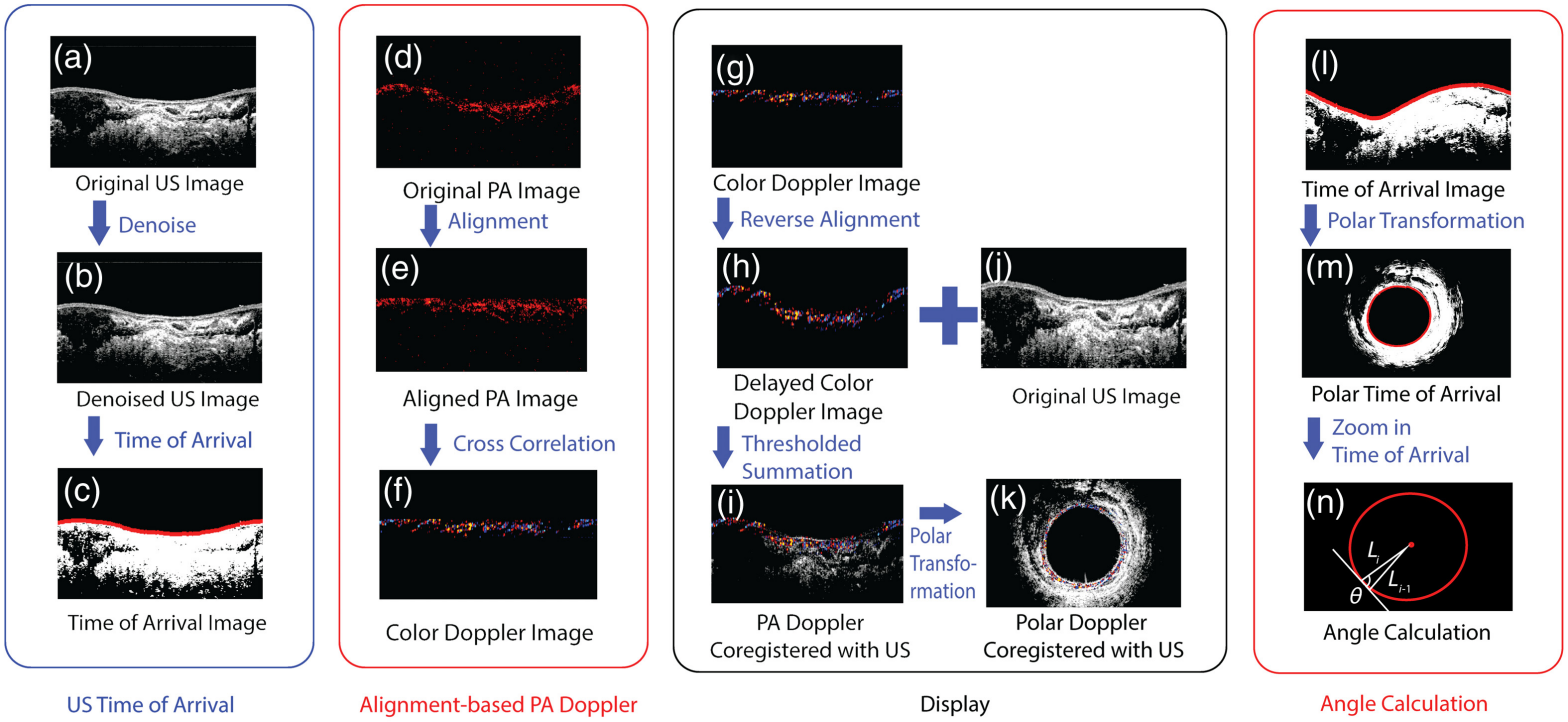
Time of arrival correction algorithm workflow and PA/US coregistration workflow. (a) The original US image is denoised at 10 dB above the noise floor to give (b). To compensate for tissue displacement, the rising edge in (c) is aligned with (d) the original PA image to yield (e) an aligned PA image, where the time of arrival is the same for all A-lines. Correlational Doppler processing yields (g). The Doppler data are subsequently backshifted (h) and coregistered with the original US image (j) to give (i) the Doppler and US coregistration image. This coregistered image is then converted to (k) polar display to better represent the scanning. (l) The rising edge time of arrival is changed to (m) polar display and used in angle calculation (n) for angle compensation.

Angle compensation is conventional in various Doppler velocimetry applications, where it is used to calculate the velocity component along the desired direction from the velocity measured along the A-line. Figure 3(b) shows the angle θ and the true versus measured velocities. We implemented angle compensation for *in vivo* images by calculating the angle between the imaging and flow direction, using the estimated time of arrival from Sec. 2.5 and the transducer surface offset of 5.57 mm, a process shown in Figs. 4(l)–4(n). The formula for the angle calculation is Eq. (3). Since the compensation factor approaches infinity as the angle between the flow direction and the scanning direction approaches 90 deg, the compensation angle is limited to 70 deg to prevent over-compensation at small angles:

$$(L_{i}+\text{offset})/\sin(\theta_{i})=(L_{i-1}+\text{offset})/\sin(180-0.36-\theta_{i-1})\;\approx (L_{i-1}+\text{offset})/\sin(180-\theta_{i-1}),$$ (3)

Equation (3) is used for calculating the angle compensation, where $\theta_{i}$ is the angle between the scanning A-line’s flow and imaging directions, $\theta_{i-1}$ is the angle between the previous scanning A-line’s flow and imaging directions, $L_{i}$, $L_{i-1}$ are the times of arrival of adjacent A-lines, offset is the distance from the transducer surface to the rotation axis.

To display the flowmetry, the velocity map is re-plotted with a color Doppler color scheme, in which black represents stationary regions, red to orange represents regions of increasing positive velocity, and blue to cyan represents regions of negative velocity with increasing speed. Finally, the color Doppler map is superimposed on the US image. For *in vivo* and spiral phantom images, the images are converted to a polar display for co-registered geometrical representation. For *in vivo* data, B scan images are uniformly divided into 20 regions, and the Doppler flow speeds in the regions are calculated for statistical analysis. One limitation of this correlational Doppler method is that it can detect flow only if absorbers move along the A-line direction. If they flow orthogonal to the A-line direction, they cannot be measured. In this research, the probe is almost always off-center, and the vasculature directions are highly variable, so the flow direction is usually not normal to the scan direction, which makes angle compensation particularly important in calculating flow velocities.

### 2.4 Phantom Imaging Setup

Figure 3(a) shows the open loop flow setup for imaging the linear flow and spiral flow phantoms. The AR-PAM/US imaging system is mounted on a manual two-dimensional (2D) translation stage to allow fine adjustment of the scanning location. Because no appropriately sized microspheres absorbing at 1064 nm were available, we produced our own contrast agent by sonicating a mixture of 60%w 50  μm glass beads infused with 40%w hydrophobic black dye. A 2%w dilution of the contrast agent was found to be optimal for PA imaging, with only moderate photothermal damage to the flow tubing. The imaging setup and scanning geometry for the linear flow phantom experiment are shown in Fig. 3(a). The imaging head is held stationary for maximum consistency in imaging the 580  μm ID PTFE tubing, and both are submerged in degassed DI water for acoustical coupling. The axis of the imaging head is held at 60 deg to the tubing bore. To study the angle dependence of flow measurements, we wrote a LabVIEW program to rotate the imaging head, allowing fine adjustment of the scanning angle.

To simulate the *in vivo* imaging conditions, we imaged the spiral flow phantom, as shown in Fig. 3(a). The AR-PAM imaging probe is mounted on a two-axis translational stage, with its imaging head submerged in degassed DI water. The processing for the spiral flow phantom is special because the imaging direction is perpendicular to the spiral flow direction, thus constant angle correction was applied. Due to tissue motion and the handheld probe consideration, our application of Doppler velocimetry employs limited averaging of 100 A-lines to the spiral flow phantom and *in vivo* data.

### 2.5 Time of Arrival Correction for *In Vivo* and Spiral Flow Phantom Data Processing

Time of arrival correction, shown in Fig. 4, compensates for shifts in the signal due to shifts in the physical location of the rectal tissue, allowing correlational Doppler to more accurately estimate shifts in the optical absorber locations. Assuming the PA and US A-lines overlap, we developed an algorithm for aligning the individual PA A-lines. Because the physical boundary between the coupling DI water and the phantom/tissue is well defined in the US image, rising edge-based US time of arrival detection was applied to the US A-lines, yielding Fig. 4(c), in which the red line represents the time of arrival. To eliminate noise points in the rising edge detection, the threshold was manually set to 10 dB above noise floor, yielding Fig. 4(b). To remove outliers, the calculated time of arrival locations were passed through an outlier rejection algorithm. Based on their times of arrival, PA A-lines are shifted in time for correlational Doppler calculations and shifted back to their original location for coregistration with the US image, as shown in Figs. 4(e)–4(i). The PA Doppler image co-registered with the US image is then warped for display in polar coordinates, as shown in Fig. 4(k).

### 2.6 *In Vivo* Imaging of Post Treatment Colorectal

For *in vivo* imaging, patients with rectal adenocarcinoma who had undergone radiation and chemotherapy were imaged pre-operatively before rectal tissue resection. Patients were first put under anesthesia per the standard of care, a colonoscopic exam was performed to localize the cancer, and our AR-PAM/US probe was then used to scan the patient’s rectum. The findings from five patients are included in this paper: three patients (P1, P2, P3) with residual adenocarcinoma, one with complete response (P4) of no remaining cancerous tissue, and one patient (P5) with a stricture below the tumor so only lesion-free tissues were imaged. This study was approved by the Institutional Review Board and was Health Insurance Portability and Accountability Act (HIPAA) compliant (ClinicalTrials.gov identifier NCT04339374), and all patients provided written informed consent.

## 3 Results

### 3.1 Linear Phantom Flow Experiment

**Fig. 5:**
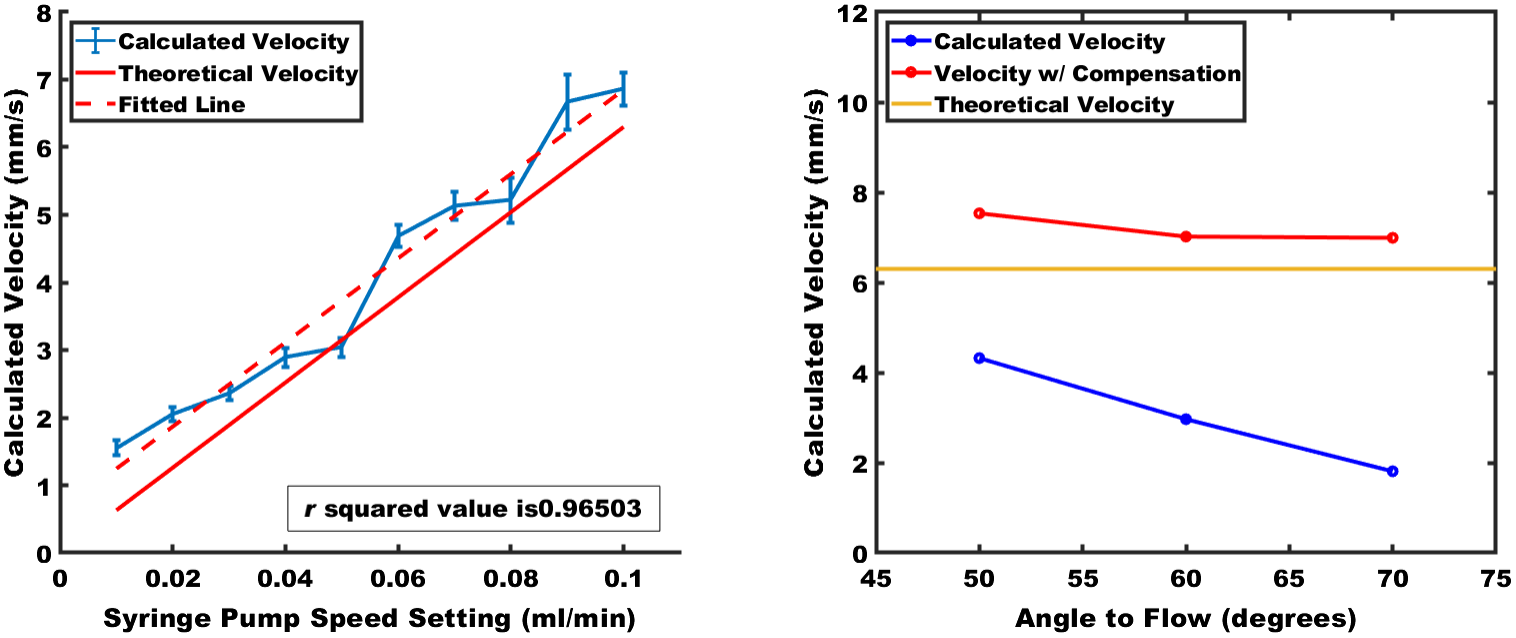
(a) Flow speed measurements from 0.01 to 0.1  ml/min. A fitted line with $R^{2}$ value of 0.965 and theoretical velocity is shown. (b) Experimental angle compensation results, where blue is the calculated velocity, red is the compensated velocity, and yellow is the actual flow velocity.

A set of flow speeds, ranging from 0.63 to 6.3 mm/s in increments of 0.63 mm/s were measured with the linear flow imaging setup to observe the flow profile. Twenty US and PA coregistered B Scans were acquired for each flow speed setting, with the results shown in Fig. 5(a). The measurements demonstrate good linearity across the flow speeds in the measurement interval, and even though a slight discrepancy between the theoretical and measured values can be observed, the differences are within 15%.

To study the effect of angular changes on the flow speed measurement, we varied the angle between the imaging head and flow direction from 70 deg to 50 deg, in 10 deg increments, while maintaining the flow speed at 6.3  mm/s. As Fig. 5(b) shows, the angle compensation procedure can compensate for large angles between the flow direction and imaging direction, but at angles <70  deg, over-compensation occurs because the compensation factor increases exponentially. Consequently, we limited the angle compensation to 70 deg.

### 3.2 Spiral Doppler Flow Experiment

**Fig. 6:**
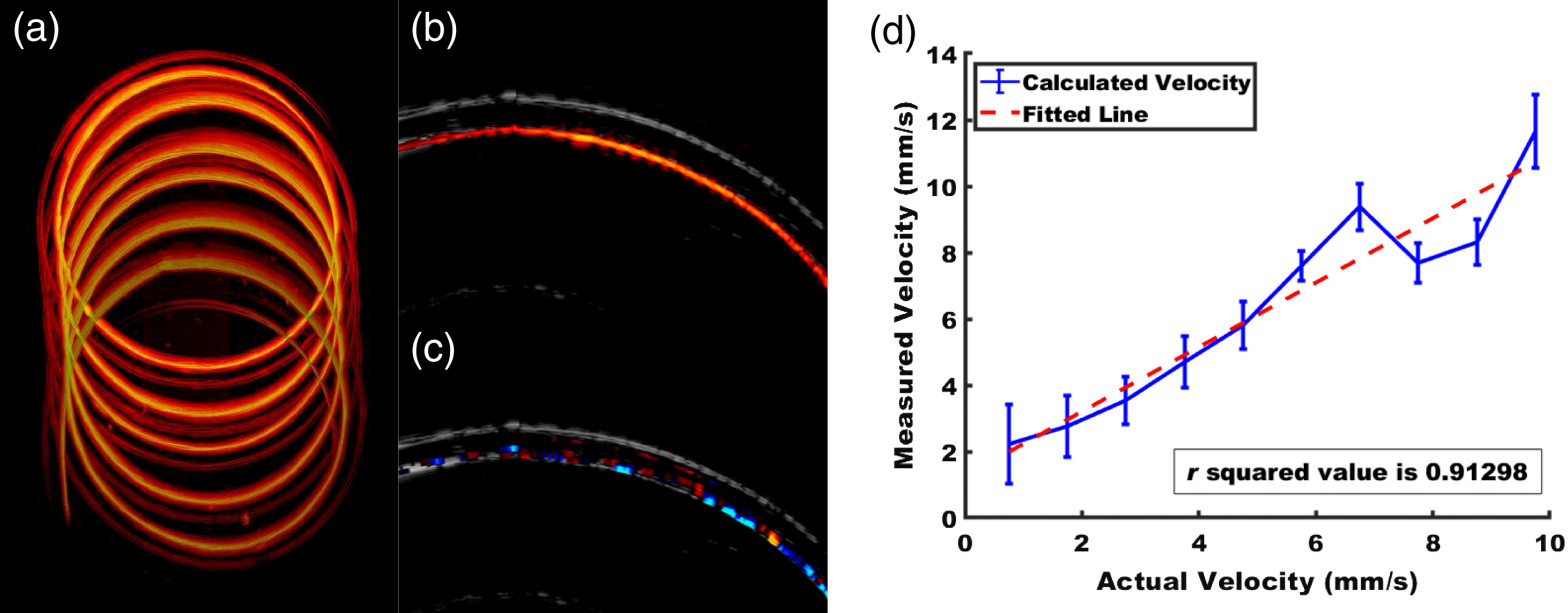
Spiral flow phantom imaging. (a) 3D AR-PAM endoscopic image of the spiral phantom. Contrast dye deposited on the inner radius of the spiral tubing wall generated a higher PA signal than dye on the outer radius. (b) PA coregistered with the US image of the scan plane that maximally intersects the tubing bore (c) Doppler image coregistered with US image. (d) Variation of Doppler-calculated flow speed with actual speed. 100 A-line ROI averaging is applied to the data, and a fitted curve with $R^{2}$ value of 0.913 is shown.

Figure 6(a) shows a 3D PA scan of the spiral flow phantom. This image allowed us to identify the turn of the tubing spiral that overlapped the most with the 2D imaging plane and consequently provided the most flow speed data. It also confirmed that the tube had no obstructions or kinks to cause flow speed irregularities. The flow speed in the spiral phantom was calculated from AR-PAM/US system data. Because the PA signals from the inner surface of the tubing are strong, as shown in Fig. 6(b), only a weak signal can be observed inside the tubing. The Doppler image coregistered with the US image, Fig. 6(c), appears quite similar, with measurable flow near the inner tubing surface. At the same time, in Fig. 6(c), we observe flow inside the tubing highlighted by Doppler processing, in contrast to the low signal amplitude in Fig. 6(b). These sets of images emphasize Doppler processing’s strength in providing perfusion information with little dependence on signal amplitude, which is valuable for *in vivo* data. Due to the difficulty of aligning the PA data with the low US signal, PA Doppler could be calculated only for regions with a strong US signal, but this should not be an issue for *in vivo* data since the water–tissue interface generates a strong US reflection.

Statistically, spiral flow signals exhibit linear trends similar to those linear flow, but with larger deviations, which is expected since only 100 A-line ROI averaging was applied to the data as compared to averaging across 1000 A-lines in Fig. 5(a), and the signal-to- noise ratio is poorer because a portion of the tube does not exhibit a strong PA signal. Still, the actual speed and estimated speed are approximately linearly correlated with an $R^{2}$ value of 0.913, demonstrating the utility of flowmetry in spiral imaging scenarios.

### 3.3 *In Vivo* Processing Results

Doppler processing was applied to *in vivo* images to test velocity estimation. Six rectal images were processed for five different patients: three patients with residual cancer (P1-3), one treatment responder (P4) with no residual cancer, and one patient (P5) with normal rectal tissue.

**Fig. 7:**
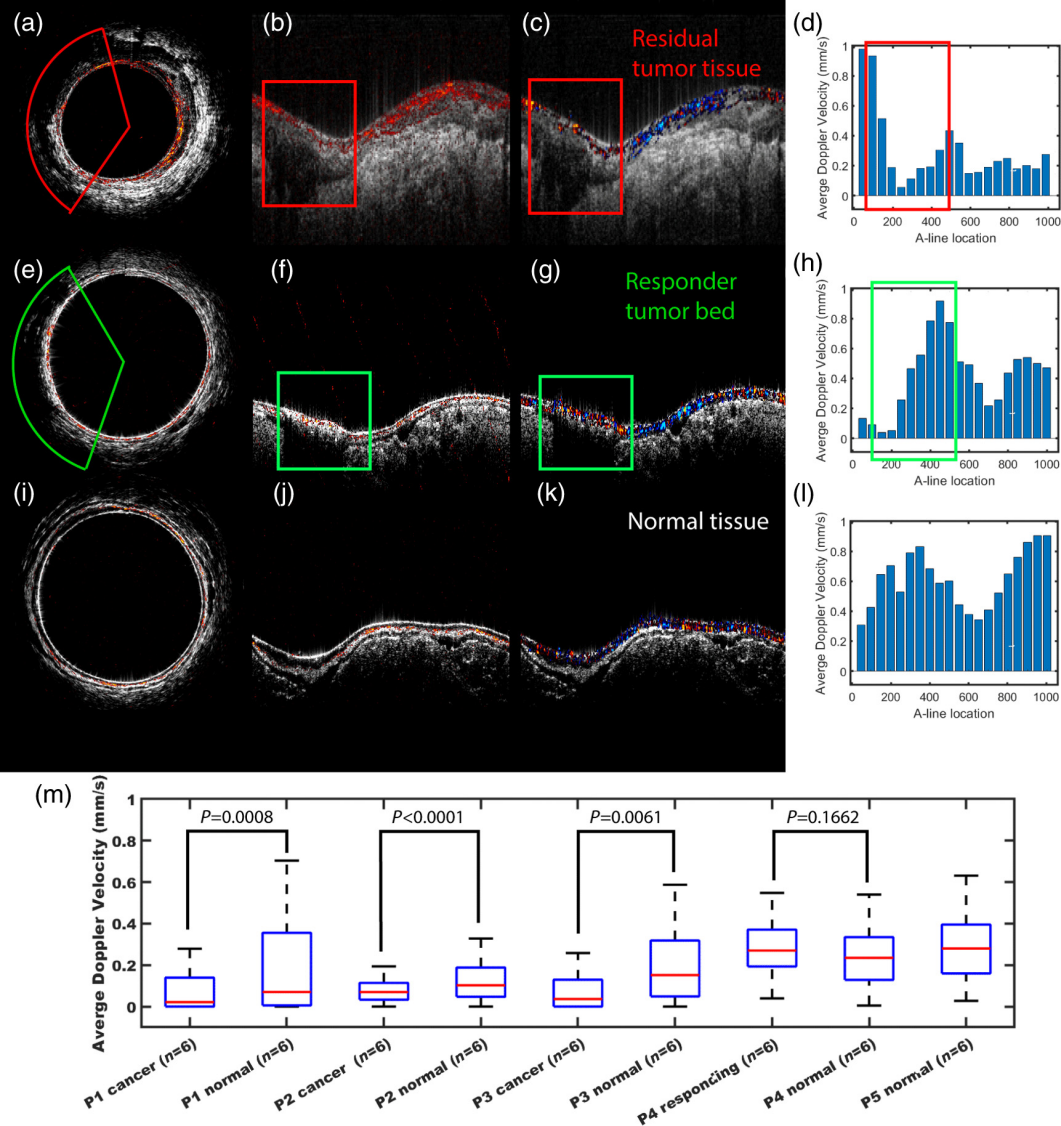
Examples of PA images coregistered with US, Doppler coregistered with US, and flow speed across a rectal cross-section. (a) Polar and (b) linear US coregistered with PA scan of a patient with cancerous tissue bordered in the red rectangle. (c) US coregistered with a Doppler scan of the same patient (d) Doppler flow speed bar chart across rectal tissue of the same patient, with cancerous tissue bordered in red. (e) Polar and (f) linear US coregistered with a PA scan of a patient with treatment scarring, bordered in green (g) US coregistered with Doppler scan of the same patient with treatment scar, labeled in green (h) Doppler flow speed bar chart across rectal tissue of patient with treatment scar, labeled in green (i) polar and (j) linear US coregistered with PA scan of patient with normal rectal tissue (k) US coregistered with Doppler scan of the patient with normal tissue (l) Doppler flow speed bar chart across rectal tissue of the same patient. (m) Box plot of average Doppler velocity across patients, analyzed with a t-test, the cancer regions exhibit statistically significant lower velocity than normal regions in all three cancer patients (P1–P3), and the responding tumor bed region exhibits slightly higher flow velocities than normal regions, but these are not statistically significant.

Figures 7(a)–7(c) show a polar scan, co-registered US and AR-PAM of a tumor bed, and a flow map of the same region, respectively, demonstrating that correlational Doppler can highlight low flow in a tumor bed (the darker regions in the red box) as distinct from the nearby higher flow regions. The residual tumor in Figs. 7(a)–7(c) displays perfusion on the surface, with no perceivable flow inside the tumor, possibly due to necrotic tissue, but higher velocity and densely packed vessels are observed adjacent to the residual tumor. In the treatment responder [Figs. 7(e)–7(g)] and normal tissue [Figs. 7(i)–7(k)], a more uniform blood flow profile across the rectal cross section is observed. For a more quantitative analysis, we averaged the Doppler flow velocity of the cancerous patients’ images in cancerous and normal regions, the treatment responder’s images in treatment scar and normal regions, and the normal patient’s images in normal regions, with a sample size of 6 for each patient and type of tissue. A Student’s t-test was used to evaluate the difference of P1 to P4’s cancerous or responding tumor bed, versus normal tissues. As shown in the box plot Fig. 7(m), there are clear statistically significant lower velocities in the cancer regions compared to normal regions in all three cancer patients. The responding tumor bed regions with scarring exhibited higher flow velocities than normal tissue regions, though not statistically significant. And even though other confounding factors including inter-patient variability and tissue heterogeneity influence flow velocities in patient rectums, the flow velocities of normal regions and responding tissue region are generally higher across all five patients.

## 4 Summary and Conclusion

In this study, we implemented acoustic resolution PA correlational Doppler flowmetry with an AR-PAM/US system. Linear and spiral flow phantoms were constructed and studied with a novel PA contrast made from glass microbeads and hydrophobic black dye. The results showed that correlational Doppler flowmetry has good linearity, its results agree well with theoretical values for linear flow and have a linear relationship with theoretical spiral flow values. A signal alignment and processing pipeline were developed for acquiring Doppler flowmetry images that are similar to conventional US Doppler images. Patient examples demonstrate correlational Doppler flowmetry’s ability to extract blood perfusion information, quantifying the expected lower flow rate inside treated cancerous tissues and relatively higher flow rate in adjacent normal tissues. In addition, the imaged differences among cancerous tissue, tumor bed tissue with no residual cancer, and normal rectal tissue suggest that flowmetry is a promising complementary modality for accurate diagnosis of residual cancer.

To our knowledge, this is the first *in vivo* application of AR-PAM Doppler flowmetry. Blood flow velocity calculated from AR-PAM data provides additional functional information to complement AR-PAM-based optical absorption imaging and improve the cancer diagnosis and cancer treatment response assessment. Similar to Doppler ultrasound, the AR-PAM Doppler mode can be turned on to quantify the local blood flow in a selected region of interest. In addition, power Doppler can be implemented, which should be more sensitive than the flow velocity estimation. Currently, the AR-PAM Doppler flow calculation is done off-line because the algorithm is computationally expensive. However, real-time flow velocity estimation and display can be done by using a computer with advanced GPU after further validation of the algorithm reported in this study. We anticipate that the AR-PAM Doppler mode will be implemented in real-time to improve colorectal cancer imaging and in other oncology applications.

One limitation of our technique is that area-based angle compensation cannot compensate for the flow directions of individual vessels. A potential solution is to implement a detailed vessel tracking algorithm to process rectal vasculature images from a 3D mechanically scanning probe, yielding the flow directions of individual blood vessels for more thorough and accurate angle compensation in computing flow velocities.

Overall, through this series of experiments, we demonstrate the potential of correlational Doppler processing in quantifying blood flow. Pilot patient data showed statistically significant increased blood perfusion around the tumor periphery and more organized and distributed flow in the treated tumor bed without residual cancer and in the normal rectal tissue. This additional blood perfusion parameter can be combined with the rectal tumor morphology seen by co-registered US and the absorption profile provided by AR-PAM to more the accurately assess treatment response and optimize treatment outcomes. Currently, we are improving the prototype system and planning to recruit a large patient cohort to validate the preliminary results.

## Data Availability

Associated code is available at https://github.com/OpticalUltrasoundImaging/Photoacoustic_dopplor_code. Data are available from the corresponding author upon reasonable request.
